# Prior Exposure to Zika Virus Significantly Enhances Peak Dengue-2 Viremia in Rhesus Macaques

**DOI:** 10.1038/s41598-017-10901-1

**Published:** 2017-09-05

**Authors:** Jeffy George, William G. Valiant, Mary J. Mattapallil, Michelle Walker, Yan-Jang S. Huang, Dana L. Vanlandingham, John Misamore, Jack Greenhouse, Deborah E. Weiss, Daniela Verthelyi, Stephen Higgs, Hanne Andersen, Mark G. Lewis, Joseph J. Mattapallil

**Affiliations:** 1 Uniformed Services University, Bethesda, MD 20814 USA; 2 National Eye Institute, National Institutes of Health, Bethesda, MD USA; 3 Bioqual Inc., Rockville, MD USA; 4 Biosecurity Research Institute, Department of Diagnostic Medicine/Pathobiology, College of Veterinary Medicine, Kansas State University, Manhattan, Kansas USA; 5 Food and Drug Administration, Silver Spring, MD USA

## Abstract

Structural and functional homologies between the Zika and Dengue viruses’ envelope proteins raise the possibility that cross-reactive antibodies induced following Zika virus infection might enhance subsequent Dengue infection. Using the rhesus macaque model we show that prior infection with Zika virus leads to a significant enhancement of Dengue-2 viremia that is accompanied by neutropenia, lympocytosis, hyperglycemia, and higher reticulocyte counts, along with the activation of pro-inflammatory monocyte subsets and release of inflammatory mediators. Zika virus infection induced detectable Dengue cross-reactive serum IgG responses that significantly amplified after Dengue-2 virus infection. Serum from Zika virus immune animals collected prior to Dengue-2 infection showed significant capacity for *in vitro* antibody dependent enhancement of Dengue-1, 2, 3 and 4 serotypes suggesting that pre-existing immunity to Zika virus could potentially enhance infection by heterologous Dengue serotypes. Our results provide first *in vivo* evidence that prior exposure to Zika virus infection can enhance Dengue infection, which has implications for understanding pathogenesis and the development of vaccines.

## Introduction

Zika virus (ZIKV) is a flavivirus transmitted by *Aedes aegypti* mosquitoes and has recently reemerged as a major public health concern worldwide^1^. ZIKV infection causes mild febrile illness in most people but has been associated with microcephaly in newborns, and Guillain-Barré Syndrome in adults. Interestingly, the reemergence of ZIKV infection geographically coincides with Dengue endemic areas in South America. Dengue virus (DENV), like ZIKV, is a flavivirus transmitted by the *Aedes* mosquitoes and causes mild, acute infection in most people. However, in a subset of people, secondary exposure to a heterologous serotype has been associated with significant enhancement of infection, that is thought to be mediated by antibodies induced during primary infection against one serotype cross reacting with another serotype of DENV. Antibody dependent enhancement (ADE) is accompanied by the release of pro-inflammatory mediators and vascular leakage leading to dengue hemorrhagic fever (DHF).

Studies have documented that antibodies induced during DENV infection cross react with ZIKV suggesting that antibody responses are induced against shared antigenic epitopes^2, 3^. Others have shown that Zika virus E protein shares ~50% sequence homology with DENV E protein^4^, and significant structural homology between ZIKV and DENV E proteins has been reported to induce conformation dependent antibody responses that were highly cross reactive^3^. The E protein is a primary target for antibody responses during DENV infection^2, 5^, and a number of ZIKV cross-reactive monoclonal antibodies were found to be specific to the DENV E protein^2^. Recent studies demonstrated that antibodies induced against ZIKV E protein significantly enhanced DENV infection *in vitro* and lethally enhanced DENV disease in mice^6^. Likewise, Kawiecki *et al*.^7^ demonstrated that polyclonal serum from mice infected with ZIKV significantly enhanced DENV replication *in vitro*. Though the potential for ZIKV immune serum to enhance DENV infection has been clearly shown in these *in vitro* studies, there is little or no evidence to date showing that pre-existing immunity to ZIKV alters the course of DENV infection *in vivo*. We sought to address this question using the rhesus macaque model for ZIKV and DENV infection^8, 9^.

## Results

### Prior infection with Zika virus significantly enhances Dengue-2 viral loads

**Figure 1:**
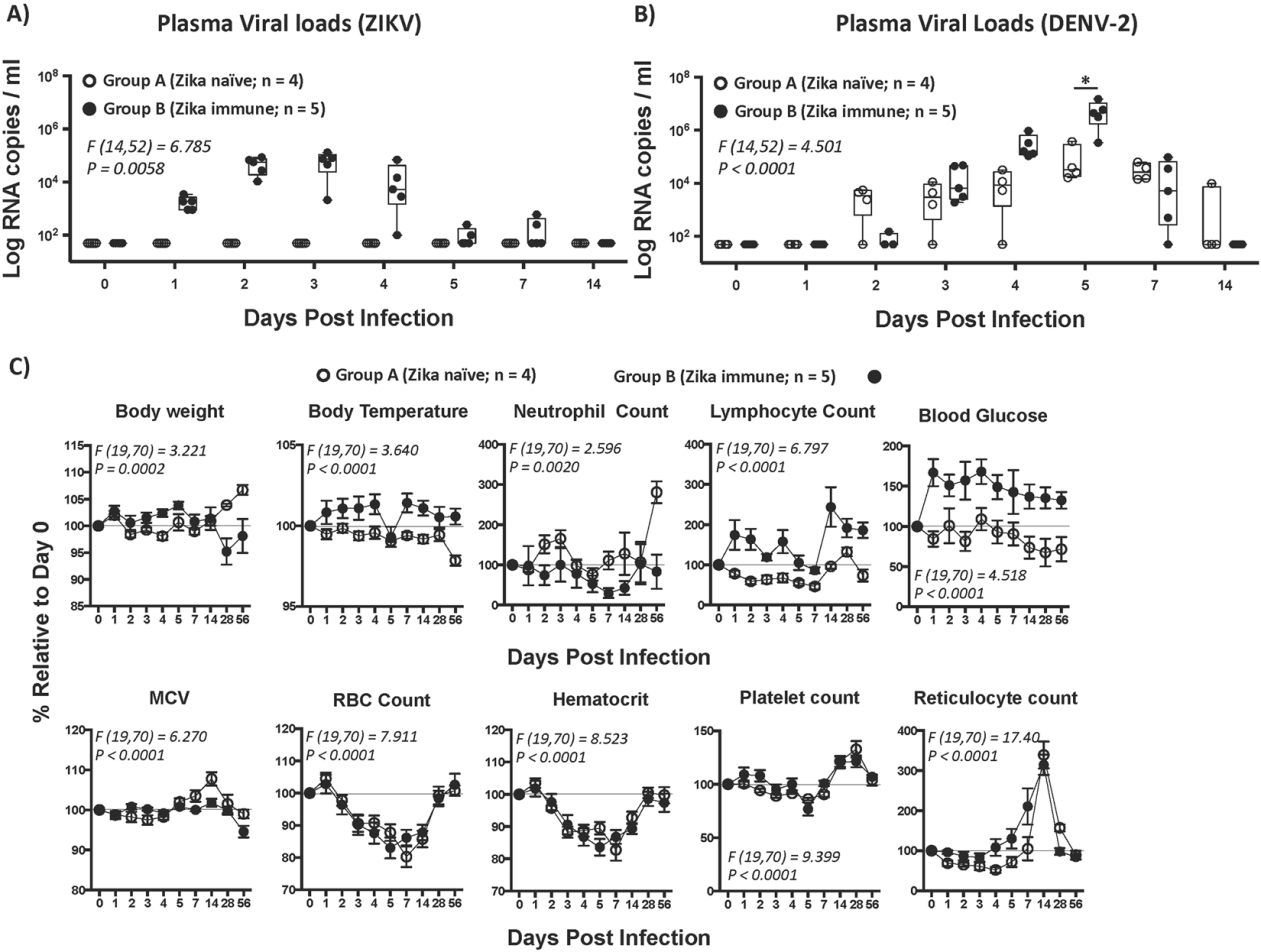
Pre-exposure to ZIKV enhances DENV-2 viremia *in vivo*. Kinetics of (**A**) ZIKV viral loads in plasma of ZIKV naïve (Group A; n = 4) and ZIKV infected (Group B; n = 5) rhesus macaques prior to DENV-2 infection. (**B**) DENV-2 viral loads in plasma from ZIKV naïve (Group A; n = 4) and ZIKV immune (Group B; n = 5) rhesus macaques after DENV-2 infection. Animals in Group A were not infected with ZIKV prior to infection with DENV-2, whereas the animals in Group B were infected with DENV-2 at day 56 post-ZIKV infection. (**C**) Analysis of body weight, body temperature, neutrophil and lymphocyte counts, blood glucose, mean cell volume (MCV), RBC, hematocrit, platelet and reticulocyte counts in blood samples that were collected longitudinally from animals in Group A (n = 4) and B (n = 5). Data are shown as % change relative to each animal’s day 0 of DENV-2 challenge values. Line represents day 0. Differences between groups were determined using One-way ANOVA and differences between time points were determined by post-hoc analysis using Tukey’s multiple comparisons test. A *p* < *0*.*05* was considered significant. Error bars represent standard error and * indicates *p* < *0*.*05*.

The kinetics of both DENV-2 and ZIKV viremia (Fig. 1A,B) were examined using plasma samples that were collected longitudinally at different time points until sacrifice. In line with earlier studies^8, 9^, ZIKV viral loads peaked at ~4–5 logs/ ml of plasma by day 3 post infection (pi) (Fig. 1A) whereas DENV-2 viral loads peaked at ~4–5 logs/ ml of plasma by day 5 pi in ZIKV naive animals (Fig. 1B). Both ZIKV and DENV-2 viral loads declined to baseline levels by 14 days pi.

In contrast to ZIKV naive animals, however, there was a significant increase in the plasma DENV-2 viral loads of ZIKV immune animals as determined by one-way ANOVA (*F* (*14*,*52*) = *4*.*501*, *p* = <*0*.*0001*) and Tukey’s post hoc analysis for multiple comparisons; ZIKV immune animals had peak DENV-2 viral loads that was ~1.5 log higher than ZIKV naive DENV-2 infected animals (Fig. 1B); mean DENV-2 peak viral load was ~113,000 copies/ml of plasma for ZIKV naïve animals whereas it was 5,747,000 copies/ml of plasma for ZIKV immune animals. The extent of increase was similar to earlier studies that examined the kinetics of DENV-2 viremia in macaques that were immune to DENV 1, 3, or 4 serotpyes^10^ raising the possibility that ZIKV likely mimics a primary DENV infection whereas DENV-2 infection in ZIKV immune animals resembled infection with a heterologous serotype. DENV-2 viremia declined to baseline levels by day 14–28 pi. ZIKV immune animals experienced a significant decrease in body weight and higher body temperatures following DENV-2 infection as compared to ZIKV naïve animals infected with DENV-2 (Fig. 1C). Enhanced viremia in ZIKV immune animals was accompanied by significant neutropenia, lympocytosis, hyperglycemia, decreased mean cell volume (MCV) along with significantly higher reticulocyte counts as compared to ZIKV naïve DENV-2 infected animals. There were no significant differences between the ZIKV naïve and immune groups of animals in other parameters examined. These findings appear to be in line with earlier studies; Onlamoon *et al*.^11^ showed that infection of rhesus macaques with the same strain of DENV-2 as used in our study was associated with severe leucopenia, neutropenia, platelet counts along with decreased hemoglobin levels and hematocrit values^11^. Similar observations have been reported in human DENV infection^12–14^. We did not observe either symptoms of rash, conjunctivitis or signs of hemorrhage in our animals.

### Enhancement of DENV-2 viral loads is associated with a significant increase in pro-inflammatory cytokine responses

**Figure 2:**
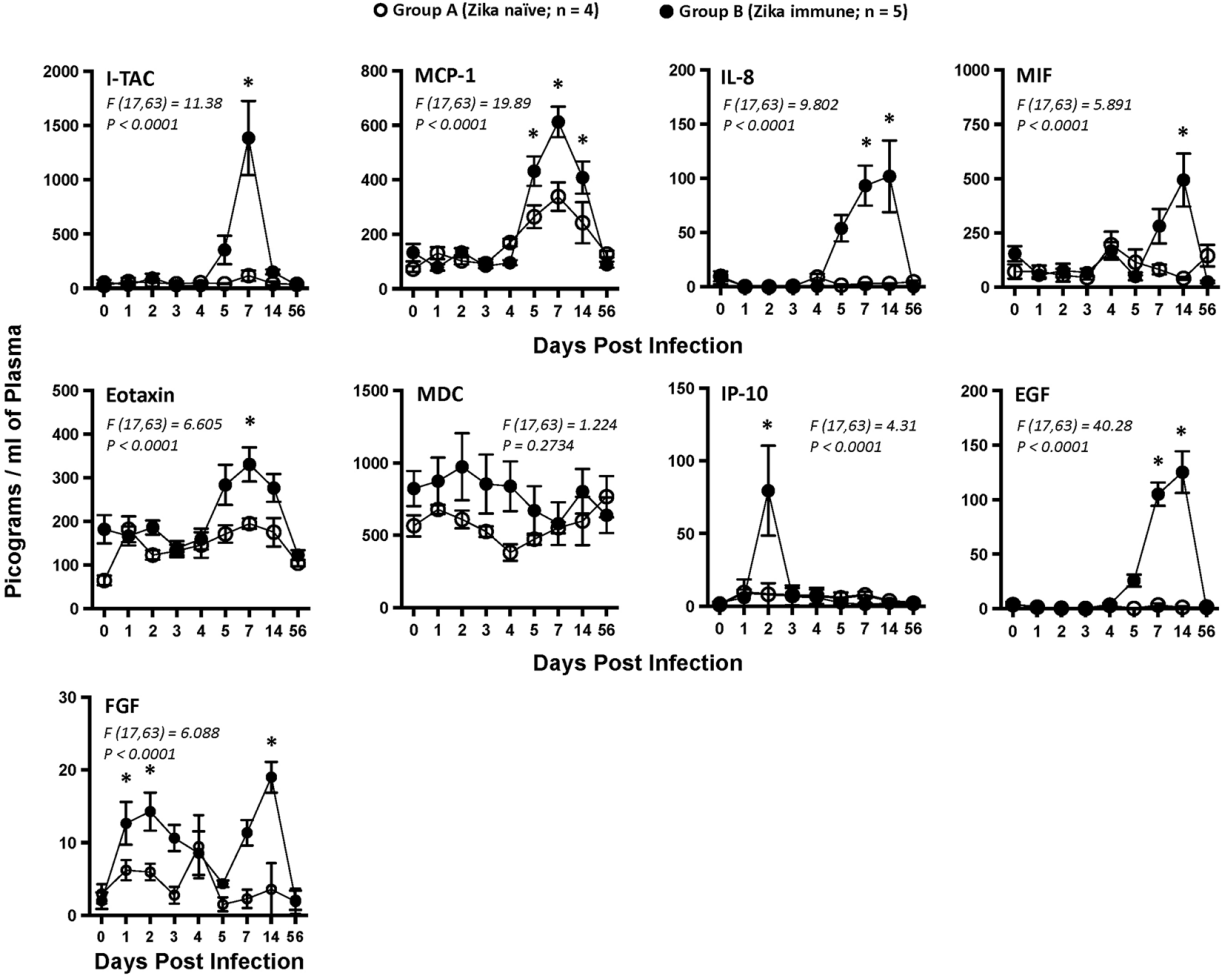
Pro-inflammatory cytokine levels are significantly upregulated in ZIKV immune animals after DENV-2 challenge. Kinetics of Interferon-inducible T-cell alpha chemoattractant (I-TAC), Monocyte chemoattractant protein-1 (MCP-1), interleukin-8 (IL-8), Macrophage inhibitory factor (MIF), Eotaxin, Macrophage derived chemokine (MDC), Interferon gamma-induced protein-10 (IP-10), Epidermal growth factor (EGF), and Fibroblast growth factor (FGF) in plasma that were collected longitudinally from ZIKV naïve (Group A; n = 4) and ZIKV immune (Group B; n = 5) rhesus macaques after DENV-2 challenge. Differences between groups were determined using One-way ANOVA and differences between time points were determined by post-hoc analysis using Tukey’s multiple comparisons test. A *p* < *0*.*05* was considered significant. Error bars represent standard error and * indicates *p* < *0*.*05*.

Next we examined if increased DENV-2 viremia in ZIKV immune animals was associated with altered levels of pro-inflammatory cytokines (Fig. 2). Previous studies have reported that enhancement of DENV infection was characterized by the release of inflammatory mediators that was associated with increased pathogenesis^15, 16^.

We observed a significant increase in plasma levels of Interferon-inducible T-cell alpha chemoattractant (I-TAC), Monocyte chemoattractant protein-1 (MCP-1), interleukin-8 (IL-8), Macrophage inhibitory factor (MIF), Eotaxin, Macrophage derived chemokine (MDC), Interferon gamma-induced protein-10 (IP-10), Epidermal growth factor (EGF), and Fibroblast growth factor (FGF) in ZIKV immune animals at day 5–14 after challenge with DENV-2 as compared to ZIKV naïve DENV-2 infected animals. The kinetics of inflammatory cytokine responses coincided with the kinetics of enhanced viremia suggesting a role for viral replication in the induction of these cytokine responses. We did not observe significant changes in other cytokines measured (Fig. S1). Studies have reported that DENV infection was associated with significant increases IL-8, MCP-1, I-TAC and numerous other cytokines^17, 18^, whereas others have reported an increase in IL-8 and MCP-1 during primary DENV-2 infection in macaques^19^. A significant increase in pro-inflammatory mediators was, however, not accompanied by overt symptoms such as rash, hemorrhage etc that are usually associated with DHF which may be related to the limitations of the rhesus macaque model as some studies have shown^10, 20–22^.

### CD14^+^ and CD14^+^CD16^+^ monocytes are significantly activated in Zika immune animals after Dengue infection

**Figure 3:**
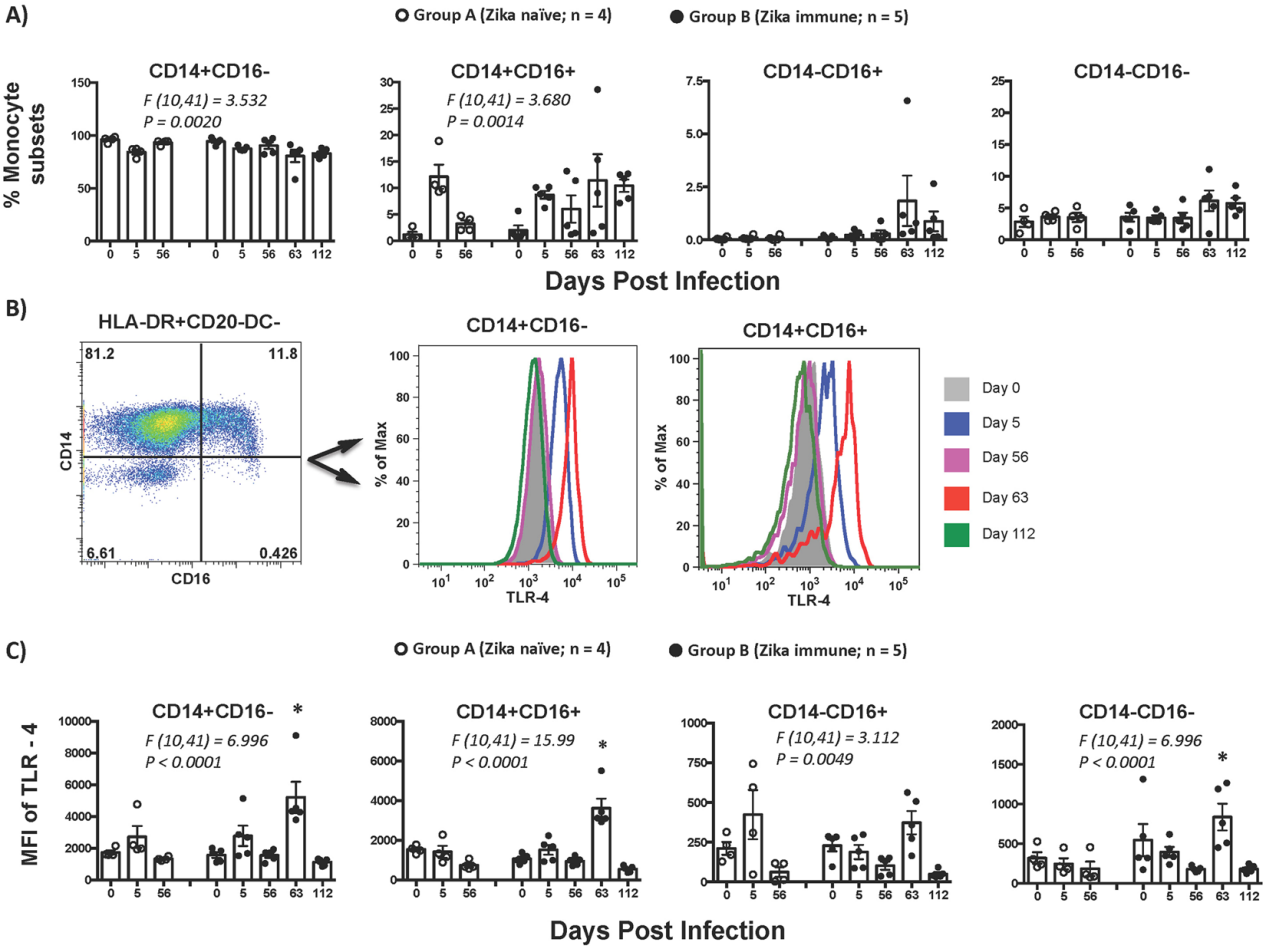
Pro-inflammatory monocyte subsets are significantly activated in ZIKV immune animals after DENV-2 infection. (**A**) Proportions of CD14^+^CD16^−^, CD14^+^CD16^+^, CD14^−^CD16^+^ and CD14^−^CD16^−^ subsets in peripheral blood from ZIKV naïve animals (Group A; n = 4) at day 0, 5 and 56 after DENV-2 challenge, and ZIKV immune animals (Group B; n = 5) at day 0, 5, 56, 63 and 112 post ZIKV infection. Day 0, 5 and 56 correspond to time points after ZIKV infection but prior to DENV-2 infection whereas day 63 and 112 correspond to Day 7 and Day 56 after DENV-2 infection. (**B**) Representative dot plots showing the discrimination of CD14 and CD16 subsets of monocytes/ macrophages, and histograms showing the level of TLR-4 expression on subsets of monocyte/macrophages in peripheral blood. (**C**) Mean fluorescence intensity (MFI) of TLR-4 expression of CD14^+^CD16^−^, CD14^+^CD16^+^, CD14^−^CD16^+^ and CD14^−^CD16^−^ subsets in peripheral blood from ZIKV naïve animals (Group A; n = 4) at day 0, 5 and 56 after DENV-2 challenge, and ZIKV immune animals (Group B; n = 5) at day 0, 5, 56, 63 and 112 post ZIKV infection. Day 0, 5 and 56 correspond to time points prior to DENV-2 infection whereas day 63 and 112 correspond to Day 7 and Day 56 after DENV-2 infection. Peripheral blood mononuclear cells that were collected longitudinally from each animal were used for analysis. Differences between groups were determined using One-way ANOVA and differences between time points were determined by post-hoc analysis using Tukey’s multiple comparisons test. A *p* < *0*.*05* was considered significant. Error bars represent standard error and * indicates *p* < *0*.*05*.

Monocyte/macrophages have been shown to be the primary target cells for DENV infection^23, 24^, and a major source of pro-inflammatory cytokines^25^. To determine if the enhancement of DENV viremia in Zika immune animals was accompanied by changes in monocyte/macrophage subsets, we examined the proportion and activation state of these cell subsets in peripheral blood samples from ZIKV immune animals that were collected longitudinally prior to and after DENV-2 infection; samples were collected at day 0, 5, 56 after ZIKV infection prior to DENV-2 infection, and at day 63 (day 7 post-DENV-2) and day 112 (day 56 post-DENV-2) after ZIKV infection and compared to ZIKV naïve DENV-2 infected animals (Fig. 3A). Monocyte/macrophage subsets were discriminated based on the expression of CD14 and CD16 on Lin^−^HLA-DR^+^CD20^−^CD11c/123^−^ myeloid cells (Fig. S2) and divided into classical (CD14^+^CD16^−^), intermediate (CD14^+^CD16^+^), non-classical (CD14^−^CD16^+^), and macrophage (CD14^−^CD16^−^) subsets as described previously^26^.

The proportions of CD14^+^CD16^−^ monocytes declined in both ZIKV naïve and ZIKV immune animals after DENV-2 infection that coincided with peak viremia (F(7,29) = 3.998; *p* = *0*.*0036*). Decline in CD14^+^CD16^−^ monocytes was accompanied by an increase in the proportions of CD14^+^CD16^+^ monocyte subsets (F(7,29) = 3.307; *p* = *0*.*0104*). These findings are in line with previous studies showing that CD14^+^CD16^−^ monocyte subsets were lower in DENV patients whereas CD14^+^CD16^+^ subsets increased as compared to healthy controls^27^. Others have shown that CD14^+^CD16^+^ monocytes significantly expanded in rhesus macaques after DENV infection^28^. There was no apparent difference in the proportion of either CD14^−^CD16^+^ or CD14^−^CD16^−^ subsets between the groups.

To assess if monocyte/macrophage subsets were activated during infection, we examined the expression of Toll-like-receptor-4 (TLR-4) on these subsets (Fig. 3B,C). Our results showed that the expression of TLR-4 was significantly upregulated on both CD14^+^CD16^−^ and CD14^+^CD16^+^ monocyte subsets, and on CD14^−^CD16^−^ subsets of macrophages in ZIKV immune animals at day 63 post-ZIKV infection (day 7 post-DENV-2) as compared to day 0 and 56 post-ZIKV infection (time points prior to DENV-2 infection) suggesting that DENV-2 infection was accompanied by a significant activation of inflammatory subsets of monocyte/macrophages. Previous studies have reported that TLR-4 expressing CD14^+^ cells significantly increased in DENV patients^27, 29^, and DENV directly activated human macrophages via TLR-4 leading to the induction and release of pro-inflammatory cytokines, and this effect was inhibited with TLR-4 antagonists, and anti-TLR-4 antibody^30^. Others have shown that non-classical monocytes (CD14^+^CD16^+^) produce significant levels for pro-inflammatory cytokines^31^.

### ZIKV immune animals display high levels of DENV cross-reactive binding antibody responses but show little neutralizing activity against DENV-2

**Figure 4:**
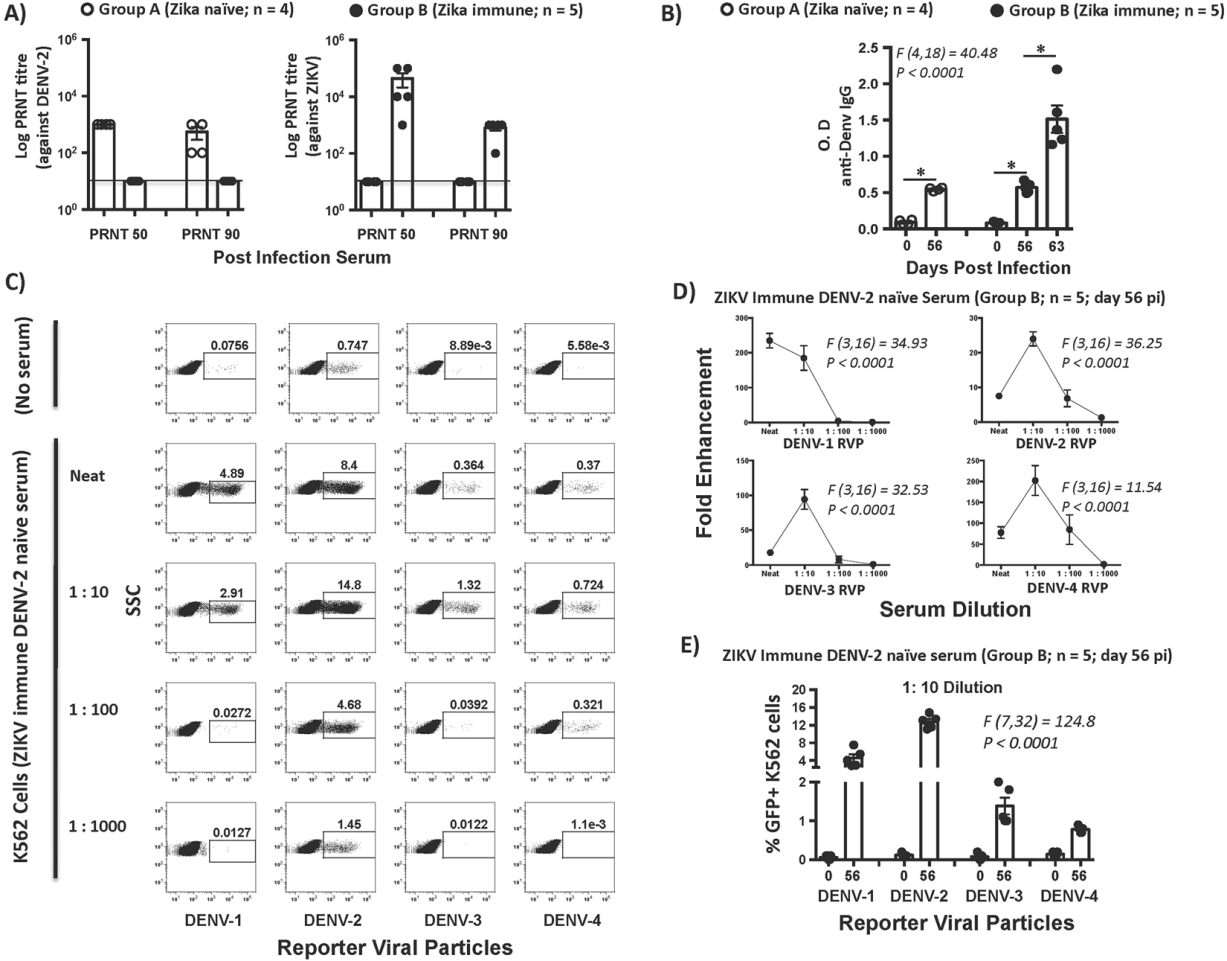
Serum from ZIKV immune animals collected prior to DENV-2 challenge significantly enhances DENV infection *in vitro* (**A**) Plaque reduction neutralization (PRNT) 50 and 90 titres against DENV-2 and ZIKV using serum from ZIKV naïve DENV-2 challenged animals (Group A; n = 4) and ZIKV immune animals (Group B; n = 5) prior to DENV-2 challenge. Line represents the limit of detection at 1:10 dilution. (**B**) Serum IgG levels in ZIKV naïve animals (Group A; n = 4) at day 0 and day 56 after DENV-2 challenge, and ZIKV immune animals (Group B; n = 5) at day 0, 5, 56 and 63 post ZIKV infection with day 0, 5 and 56 corresponding to time points prior to DENV-2 infection whereas day 63 corresponding to Day 7 after DENV-2 infection. Group B animals were infected with DENV-2 at day 56 after ZIKV infection. Serum samples collected longitudinally were used for analysis. (**C**) Representative FACS plots showing infection of K562 cells with DENV-1, 2, 3 and 4 reporter viral particles (RVP) using day 56 serum from ZIKV immune animals (Group B; n = 5) collected prior to DENV-2 challenge. K562 cells were incubated with either neat or serially diluted serum at 1:10, 1:100, and 1:1000 dilution in the presence of RVP’s and GFP expression was examined by flow cytometry. (**D**) Fold antibody dependent enhancement of DENV-1, 2, 3 and 4 RVP infection of K562 cells using serum from ZIKV immune animals (Group B; n = 5) that was collected at day 56 after ZIKV infection but prior to DENV-2 challenge and (**E**) Frequency of RVP + K562 cells infected with DENV-1, 2, 3 and 4 RVP at 1: 10 dilution of serum from ZIKV immune animals (Group B; n = 5) that was collected at day 56 after ZIKV infection but prior to DENV-2 challenge. Differences between groups were determined using One-way ANOVA and differences between time points were determined by post-hoc analysis using Tukey’s multiple comparisons test. A *p* < *0*.*05* was considered significant. Error bars represent standard error and * indicates *p* < *0*.*05*.

Previous studies have shown that subneutralizing concentrations of DENV specific antibodies enhanced DENV infection in humans^32^. To determine if ZIKV infection induced DENV-2 cross-reactive neutralizing antibody (nAb) responses, we examined the potential of serum collected from ZIKV immune animals prior to infection with DENV-2 (Group B; day 56 post-ZIKV infection), and from ZIKV naïve DENV-2 infected animals (Group A; day 56 post-DENV-2 infection) to neutralize ZIKV and DENV-2 using plaque reduction neutralization antibody (PRNT) assays (Fig. 4A). Pre-ZIKV (Group B) and pre-DENV-2 (Group A) serum were used as negative controls.

As expected, we observed high PRNT_50_ and PRNT_90_ nAb titres against DENV-2 but not against ZIKV (PRNT_50/90_ = or <10) in the ZIKV naïve DENV-2 challenged animals (Group A). Likewise, ZIKV immune animals (Group B) displayed significantly high nAb responses against ZIKV but not against DENV-2 (PRNT_50/90_ = or <10) suggesting that ZIKV infection induced either little or no detectable DENV-2 cross-reactive nAb responses at the time points examined. These findings are in line with previous studies showing little or no DENV-2 specific nAb titres (PRNT_90_ = or <10) in primary infection with ZIKV^33^. Likewise, Collins *et al*.^34^ recently showed that little or no durable cross neutralizing antibodies against ZIKV were induced by DENV infection.

To determine if ZIKV infection induced high levels of cross-reactive non-nAb responses against DENV, we examined the relative levels of DENV specific IgG in serum from both ZIKV naïve DENV-2 immune (Group A; day 0 and 56 post DENV-2) and ZIKV immune animals (Group B; day 0 and 56 pi prior to DENV-2 infection and at day 7 after DENV-2 infection) using a DENV specific ELISA assay for serum IgG^35^. Significant levels of anti-DENV IgG were readily detectable in the serum of both groups of animals (Fig. 4B) suggesting that ZIKV infection induced significant levels of anti-DENV specific cross-reactive antibodies. The levels of DENV specific IgG was in line with what has been previously reported in DENV-2 infected rhesus macaques at the similar time points after infection^19^. Anti-DENV IgG titres significantly increased in ZIKV immune animals at day 7 after challenge with DENV-2 (Fig. 4B; day 63 post ZIKV infection) suggesting that DENV infection was associated with a significant expansion of DENV-2 cross-reactive anamnestic B cell responses.

### Serum from ZIKV immune animals display significant *in vitro* capacity to enhance infection by heterologous DENV serotypes

Previous studies have shown that polyclonal serum from ZIKV infected mice significantly enhanced DENV-2 replication *in vitro* ^7^ whereas Stettler *et al*.^6^ reported that antibodies induced against ZIKV E protein though poorly neutralizing significantly enhanced DENV infection in mice. To assess if the serum from ZIKV immune animals was capable of mediating ADE, we tested the potential of serum collected from ZIKV immune animals prior to DENV-2 challenge (Group B; day 56 post ZIKV infection) to enhance DENV infection *in vitro* using K562 cells, an Fc receptor expressing leukemia cell line^36, 37^, along with DENV reporter virus particles (RVP)^38^.

The DENV RVPs are structurally intact Dengue virions that are capable of only a single round of replication and encode a sub-genomic GFP replicon that is expressed following infection of target cells^38^. The RVPs do not readily infect K562 cells in the absence of DENV specific antibodies. RVPs were obtained from Integral Molecular (Philadelphia, PA) for use in ADE assays. Serum was serially diluted 10-fold and incubated with DENV-1, 2, 3 and 4 RVPs prior to culture with K562 cells. Cells were harvested after 48 hours and the frequency of GFP expressing cells were determined by flow cytometry (Fig. 4C). Concentration of RVP’s was titrated using ZIKV postive serum and K562 cells (Fig. S3). No serum and cells only controls were included in each experiment.

Our results showed that the frequency of K562 cells expressing DENV-1, 2, 3 and 4-GFP were significantly higher in the presence of ZIKV immune serum (Fig. 4D,E) suggesting that ZIKV infection induced cross-reactive antibodies against all four DENV serotypes. The proportion of K562 cells infected with DENV-1 and 2 RVPs that expressed GFP were significantly higher than DENV-3 and 4 RVPs suggesting that ZIKV infection likely induced antibodies that preferentially cross-reacted with DENV-1 and 2 serotypes as compared to the other two serotypes (Fig. 4E). Interestingly, except for DENV-1, the fold enhancement was highest at 1:10 dilution of serum (Fig. 4D), as compared to either neat or lower serum dilutions, suggesting that sub-concentrations of antibodies equally enhanced infection of DENV-2, 3 and 4 serotypes.

## Discussion

ZIKV is an emerging pathogen that is circulating in DENV endemic areas. Structural similarities between ZIKV and DENV E proteins raises the concern that cross-reactive antibody responses induced by ZIKV especially in DENV naïve populations could potentially lead to more severe pathogenic outcomes following primary infection with DENV. Our findings provide *in vivo* evidence that prior exposure to ZIKV significantly enhances primary DENV-2 viremia that was accompanied by leucopenia, neutropenia, increased reticulocyte counts, high blood glucose and thrombocytopenia, changes that are usually associated with severe DENV infection. These changes were accompanied by a significant activation of monocyte subsets and secretion of pro-inflammatory cytokines, a majority of which chemoattract monocyte/macrophages. Numerous studies have demonstrated a key role for activated monocyte/macrophages in Dengue pathogenesis^13, 25^.

ZIKV infection induced high levels of DENV cross-reactive antibodies with little or no neutralizing activity against DENV-2. This was, however, not surprising as previous studies have reported little or no DENV-2 specific nAb titres during primary infection with ZIKV^33^. Serum from ZIKV infected animals were, however, found to induce significant *in vitro* ADE of DENV-2 suggesting that the *in vivo* enhancement of DENV-2 viremia we observed may be mediated by non-neutralizing or sub-neutralizing concentrations of DENV-2 cross-reactive antibody responses. Interestingly, these antibody responses were found to enhance infection of other heterologous serotypes such as DENV- 1, 3 and 4 *in vitro* suggesting that pre-existing ZIKV specific immune responses could potentially enhance infection with all serotypes of DENV. Additional studies are needed to determine if prior exposure to ZIKV enhances infection with DENV- 1, 3 and 4 *in vivo*. Though it is difficult to determine the exact mechanisms that may have contributed to the *in vivo* enhancement of DENV-2 viremia, out results suggest that ADE could have played a role.

Though the ZIKV exposed animals experienced significant enhancement of viremia and release of pro-inflammatory mediators, why they did not display symptoms associated with DHF is not clear. There is no prior evidence of enhanced viremia with Dengue infection in rhesus macaques except for one study that reported significantly high plasma viremia and symptoms of hemorrhage in rhesus macaques that were challenged with a high dose of DENV-2 intravenously^11^. These animals experienced changes in CBC and serum chemistry similar to what we observed in our studies even though the animals in our studies were challenged with a lower dose of DENV-2 subcutaneously. It is probable that the absence of DHF like symptoms is due to the limitation of the NHP model as has been previously reported^39^. Though DHF was not observed in our studies, the significant differences between ZIKV naïve and immune animals after DENV-2 challenge suggests that prior exposure to ZIKV has the potential to enhance DENV associated pathogenesis.

Additional and larger studies are needed to determine if the changes we observed in ZIKV immune animals can be recapitulated with other strains of DENV-2, and if similar enhancement of DENV-2 occurs when ZIKV immune animals are infected with DENV-2 after longer periods of convalescence than the 8 weeks used in our study. Likewise, further clinical and translational studies are essential to better understand the effect of prior ZIKV infection on the course of DENV pathogenesis in human subjects. In conclusion, the data reported here have implications for understanding the pathogenesis of both ZIKV and DENV infections especially in DENV naïve populations, and will aid in the development of better vaccines that can protect from these infections.

## Materials and Methods

### Animals, infection and samples

Nine rhesus macaques of Indian origin acquired by Bioqual Inc. (Rockville, MD) were used in this study. All animals were seronegative for ZIKV and DENV. Animals were housed at Bioqual and cared for in accordance with local, state and federal policies in an Association for Assessment and Accreditation of Laboratory Animal Care International (AAALAC)-accredited facility. All animal experiments were reviewed and approved by Institutional Animal Care and Use Committee at Bioqual Inc., and samples were acquired through a tissue sharing protocol. The animals were divided into two groups; Group A (n = 4) was infected with only DENV-2 subcutaneously and sacrificed at day 56 post infection, and Group B (n = 5) were first infected with ZIKV subcutaneously for a period of 56 days and then infected with DENV-2 subcutaneously at day 56 post-ZIKV infection and sacrificed at day 56 post-DENV-2 infection (day 112 post-ZIKV infection). Animals were infected subcutaneously with 10^6^ TCID_50_/ml of Zika virus (Puerto Rico Strain; Genbank KU501215) and 10^5^ TCID_50_/ml of DENV-2 virus (strain 16681). The challenge titer was based on previous studies^9, 19^.

Peripheral blood samples were collected longitudinally from animals in Group A and B at day 0, 1, 2, 3, 4, 5, 7, 14, 28 and 56. Additional samples were obtained from animals in Group B at day 57, 58, 59, 60, 61, 63, 70, 84, and 112 following DENV-2 infection. Peripheral blood mononuclear cells (PBMC) were obtained by density gradient centrifugation and cryopreserved along with plasma and serum at each time point. Cumulative Blood Counts (CBC) and analysis of serum chemistry was performed at IDEXX Laboratories, Inc. (Rockville, MD).

### Absolute quantification of plasma viral loads by qRT-PCR

Plasma viral loads were determined using real-time quantitative RT-PCR assay. RNA was extracted from the plasma using the QIAamp MinElute Virus spin kit (Qiagen) and reverse transcribed using a mixture of random hexamers and anchored oligo-dT primers. Synthesized cDNA was PCR amplified using Zika^40^ (forward: GGAAAAAAGAGGCTATGGAAATAATAAAG, reverse: CTCCTTCCTAGCATTGATTATTCTCA, probe: AGTTCAAGAAAGATCTGGCTG) and DENV-2 (forward: CAGGGTGTGGATTCAAGAAAACCCATGG, reverse: TGCTTGTTAACCCAATCAATGAGCC, probe: ACTCCAGTG/ZEN/GAATCATGGGAGGAAATCCCA) specific primers and probes. The PCR reaction was set up in triplicate using Taq-polymerase (Bioline USA, Inc., (Taunton, MA) and assayed in the 7500 Taqman instrument (Applied Biosystems) using the following conditions: 48 °C for 30 minutes, 95 °C for 10 minutes followed by 40 cycles of 95 °C for 15 seconds and 1 minute at 60 °C. The number of ZIKV and ﻿DENV-2﻿ viral copies was determined using standards generated from RNA that was extracted from the ZIKV and DENV-2 viral stocks. Viral RNA was quantified and diluted at a concentration of 10^7^ copies and reverse transcribed as described above. Reverse transcribed cDNA was serially diluted to generate a standard curve ranging from 1 copy to 10^6^ copies/reaction. The limit of detection was 50 copies/ml.

### Antibody dependent enhancement assay with DENV reporter viral particles

ADE assays were performed using serum from ZIKV immune animals along with DENV-1, 2, 3, and 4 Reporter Virus Particles (RVP). RVPs^38^ are replication incompetent viral particles that express GFP after a single round of replication and were obtained from Integral Molecular Inc., (Philadelphia, PA). The serum was heat inactivated at 56 °C for 30 minutes, serially diluted 10 fold, and mixed with 10 uL of RVP at a ratio of 1:1. After incubation for 1 hour at 37 C, 5% CO2, the serum RVP mix was added to ~80,000 K562 cells in 30 ul of RPMI-10 and incubated for 1 hour at 37 °C, 5% CO2. After 1 hour, the cells were washed twice with RMPI-10 to remove unbound virus, and resuspended in RPMI-10 and cultured in a 96 well tissue culture plate for 48 hours. After culture, cells were washed in 1x PBS and fixed in 0.5% Paraformaldehyde and analyzed using an LSR II flow cytometer. Collected data was analyzed using Flowjo 9.6 software.

### Cytokine levels in plasma

Cytokine levels were determined using plasma that was collected longitudinally from each animal. Plasma cytokine levels were examined using the Cytokine Monkey Magnetic 29-Plex Panel for Luminex™ Platform (Thermofisher Scientific, Waltham, MA). This kit allows simultaneous quantification of 29 cytokines namely, FGF-basic, IL-1β, G-CSF, IL-10, IL-6, IL-12, RANTES, Eotaxin, IL-17, MIP-1α, GM-CSF, MIP-1β, MCP-1, IL-15, EGF, IL-5, HGF, VEGF, IFNγ, MDC, I-TAC, MIF, IL-1RA, TNFα, IL-2, IP-10, MIG, IL-4 and IL-8. Briefly, plasma samples were diluted 1:2 in assay diluent and used as per manufacturers instructions. The assay was repeated for each sample and the average concentration was determined for each cytokine. Plates were analyzed using Luminex xMAP technology on a Bio-plex 200 system (Biorad). Collected data was analyzed, and concentrations were determined using Bioplex manager software 6.1. The operator was blinded to the identity of the samples prior to each assay and data was unblinded after analysis.

### Antibodies and flow cytometry

PBMC were labeled with a panel of anti-CD3-Pacific blue (PB), CD8-PB, VIVID live dead stain, CD20-BV650, CD14-FITC, CD11c-PE, CD123-PE, HLA-DR-ECD, CD16-Cy-7-APC and TLR-4-APC. Monocyte subsets were discriminated based on the differential expression of CD14 and CD16 on CD3/CD8/VIVID^−^HLA-DR ^+^ CD20^−^CD11c/123^−^ myeloid cells. All the antibodies were titrated using rhesus macaque PBMC. Labeled cells were washed and fixed in 0.5% PFA and analyzed on a LSR-II flow cytometer. One million total events were collected for analysis. Collected data was analyzed using Flowjo 9.6 software. The operator was blinded to the identity of the samples and data was unblinded after analysis.

### Plaque reduction neutralization test

PRNT assays were performed using serum that was collected longitudinally. Briefly, 24-well plates were set to 100% confluence with Vero cells. Serum was heat inactivated for 30 minutes at 56 °C in a water bath. After initially diluting the serum at 1:5 in DMEM with 1% FBS, and it was diluted serially at a ratio of 1:10. ZIKV or DENV-2 viral stocks were quick thawed at 37 °C and diluted in DMEM containing 1% FBS to a titer of 4 × 10^2^ plaque forming units (pfu)/ml. An equal volume of virus was added to each dilution of serum, mixed, and incubated for 1 hour at 37 °C. After aspirating the media 75 uL of virus and serum mix containing ~30 pfu/well of virus was added to a 24 well plate. Each dilution was assayed in duplicate. After incubating the plates at 37 °C in 5% CO2 for 1 hour, 1 ml of 2x EMEM containing 10% FBS mixed with an equal volume of 2% methylcellulose, warmed to 37 C, was added to each well. The plates were incubated for a period of 5 days for ZIKV and 6 days for DENV-2 at 37 C, 5% CO2. At the end of incubation, the plates were fixed by adding 10% formalin to each well, and stained with hematoxylin and eosin and the number of plaques were counted. Percentage neutralization was determined using the formula: (number of plaques in dilution of interest)/(number of plaques in virus only control well ∗ 100). The operator was blinded to the identity of the samples prior to each assay.

### DENV specific IgG ELISA

Serological assays for DENV specific IgG were performed as per manufactures instructions (IBL-America, Minneapolis, MN) using serum that was collected longitudinally. Briefly, serum samples were diluted 1:21 in sample diluent and dispensed into the microwell strips. Negative control, positive control and calibrator were used as provided with the kit. Assay diluent alone instead of serum was used as reagent blanks. All the samples and controls were setup in duplicates. After incubating for 20 minutes, the microwell strips were washed 3x with wash buffer, and 100 ul of the enzyme conjugate was added to each well. The microwell strips were incubated for 20 minutes, washed 3x with wash buffer and 100ul of TMB substrate was added to each well. The reaction was stopped after 10 minutes using the stop buffer and the O.D was determined at 450 nm using an ELISA plate reader. The operator was blinded to the identity of the samples prior to each assay and data was unblinded after analysis.

### Data analysis

Statistical analysis was performed using GraphPad Prism Version 5.0 software (GraphPad Prism Software, Inc. San Diego, CA). Differences between groups were determined using One-way ANOVA and differences between time points were determined by post-hoc analysis using Tukey’s multiple comparisons test. A *p* < *0*.*05* was considered significant. Error bars represent standard error. All data generated during this study are included in the manuscript and available on request.

## Electronic supplementary material

Supplementary Information
