# Supplementary material for: Prior Exposure to Zika Virus Significantly Enhances Peak Dengue-2 Viremia in Rhesus Macaques
Source: Sci Rep. 2017 Sep 5;7:10498. doi: 10.1038/s41598-017-10901-1 (PMC5585353; doi:10.1038/s41598-017-10901-1)
Supplement: Supplementary file 1 — Supplementary Information [file 41598_2017_10901_MOESM1_ESM.pdf]

Prior Exposure to Zika Virus Significantly Enhances Dengue-2 Viremia in Rhesus Macaques

Jeffy George[1#], William G. Valiant[1#], Mary J. Mattapallil[2], Michelle Walker[3], Yan-Jang S. Huang[4], Dana L. Vanlandingham[4], John Misamore[3], Jack Greenhouse[3], Deborah E. Weiss[3], Daniela Verthelyi[5], Stephen Higgs[4], Hanne Andersen[3], Mark G. Lewis[3], & Joseph J. Mattapallil[1*]

[1]Uniformed Services University of the Health Sciences, Bethesda, MD 20814; [2]National Eye Institute, National Institutes of Health, Bethesda, MD; [3]Bioqual, Rockville, MD; [4]Biosecurity Research Institute, Department of Diagnostic Medicine/Pathobiology, College of Veterinary Medicine, Kansas State University, Manhattan, Kansas; [5]Food and Drug Administration, Silver Spring, MD;

Fig. S1. Plasma cytokine Levels. Kinetics of IL-1β, G-CSF, IL-10, IL-6, IL-12, IL-17, MIP-1α, GM-CSF, MIP-1β, IL-15, HGF, VEGF, IFNγ, IL-1RA, TNFα, MIG, IL-4, RANTES, IL-5, and IL-2 in plasma that were collected longitudinally from ZIKV naïve (Group A; n = 4) and ZIKV immune (Group B; n = 5) rhesus macaques after DENV-2 challenge. No significant differences were observed between the groups. Differences between groups were determined using One-way ANOVA and differences between time points were determined by post-hoc analysis using Tukey's multiple comparisons test. A $p < 0.05$ was considered significant. Error bars represent standard error and * indicates $p < 0.05$.

Fig. S2. Gating strategy used to discriminate monocyte/macrophage subsets in peripheral blood. Peripheral blood mononuclear cells (PBMC) were stained with a panel of markers to discriminate $CD3^{-}CD8^{-}VIVID^{-}HLA\text{-}DR^{+}CD20^{-}CD11c/CD123^{-}$CD14 and CD16 subsets.

Fig. S3. Titration of DENV - 1, 2, 3 and 4 Reporter viral particles (RVP) using K562 cells. The concentration of DENV RVP to be used in the antibody dependent enhancement of infection is K562 cells was determined using ZIKV positive serum. Heat inactivated serum was incubated with different concentrations of RVP and then cultured with K562 cells for 48 hours. The frequency of GFP expressing cells was determined by flow cytometry.

Fig. S4. Changes in body temperature during DENV-2 infection. Body temperatures were measured in ZIKV naïve and ZIKV immune animals after DENV-2 infection. Raw values are shown.

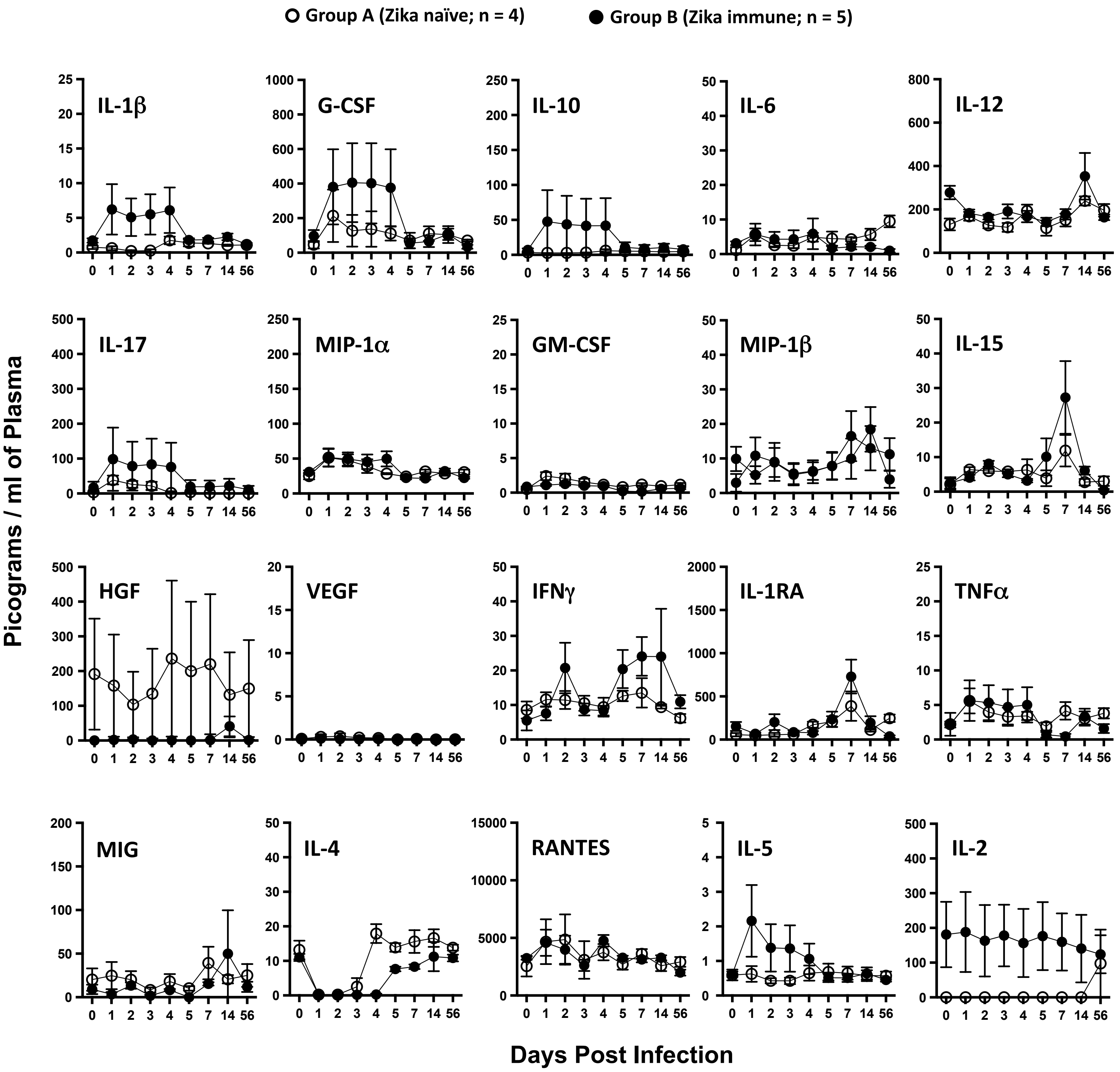
O Group A (Zika naïve; n = 4)
● Group B (Zika immune; n = 5)
IL-1β
G-CSF
IL-10
IL-6
IL-12
IL-17
MIP-1α
GM-CSF
MIP-1β
IL-15
HGF
VEGF
IFNγ
IL-1RA
TNFα
MIG
IL-4
RANTES
IL-5
IL-2
Picograms / ml of Plasma
Days Post Infection

Fig. S1

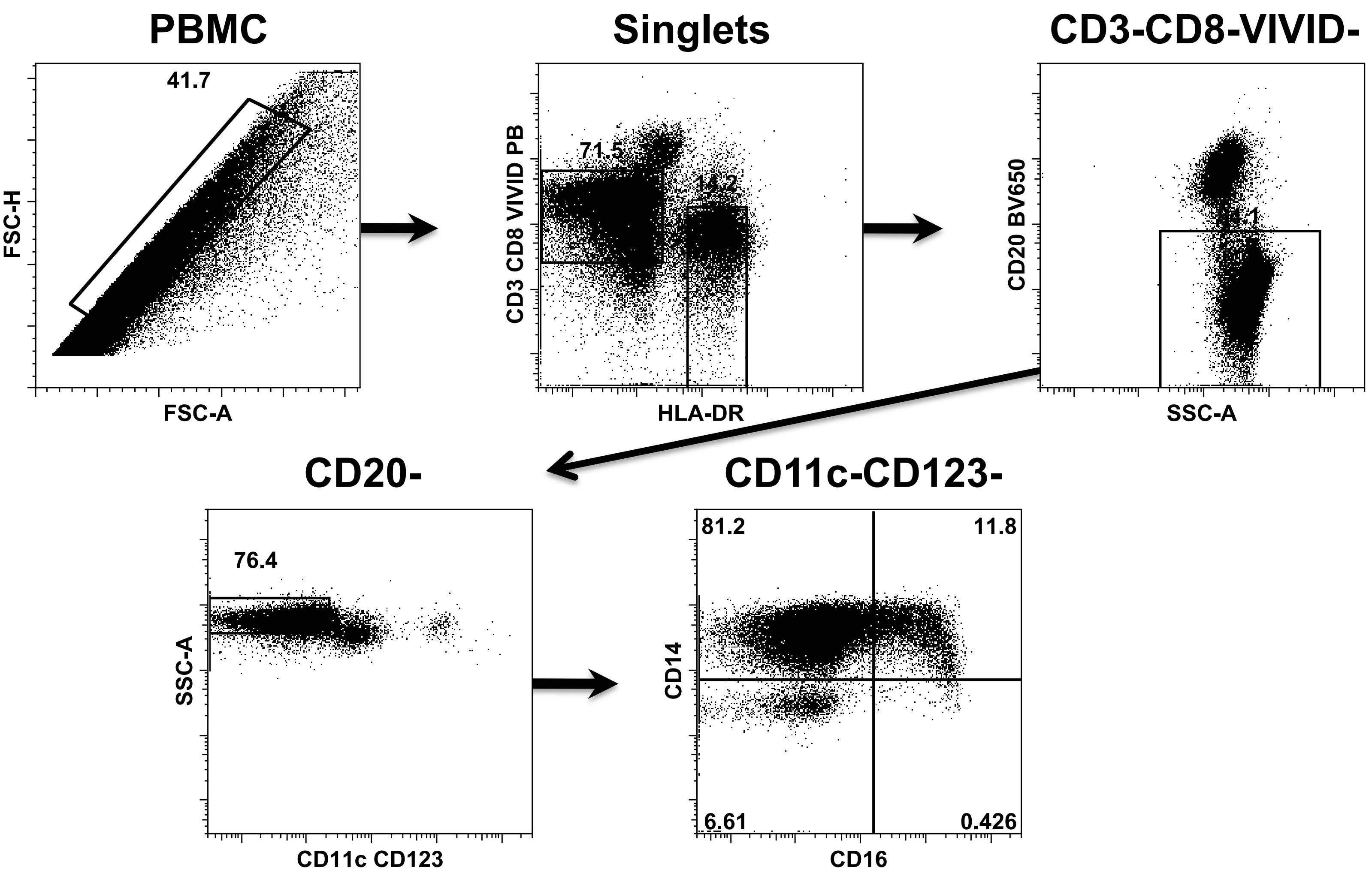
PBMC
41.7
FSC-H
FSC-A
Singlets
71.5
CD3 CD8 VIVID PB
HLA-DR
CD3-CD8-VIVID-
CD20 BV650
SSC-A
CD20-
76.4
SSC-A
CD11c CD123
CD11c-CD123-
81.2
11.8
6.61
0.426
CD14
CD16

Fig. S2

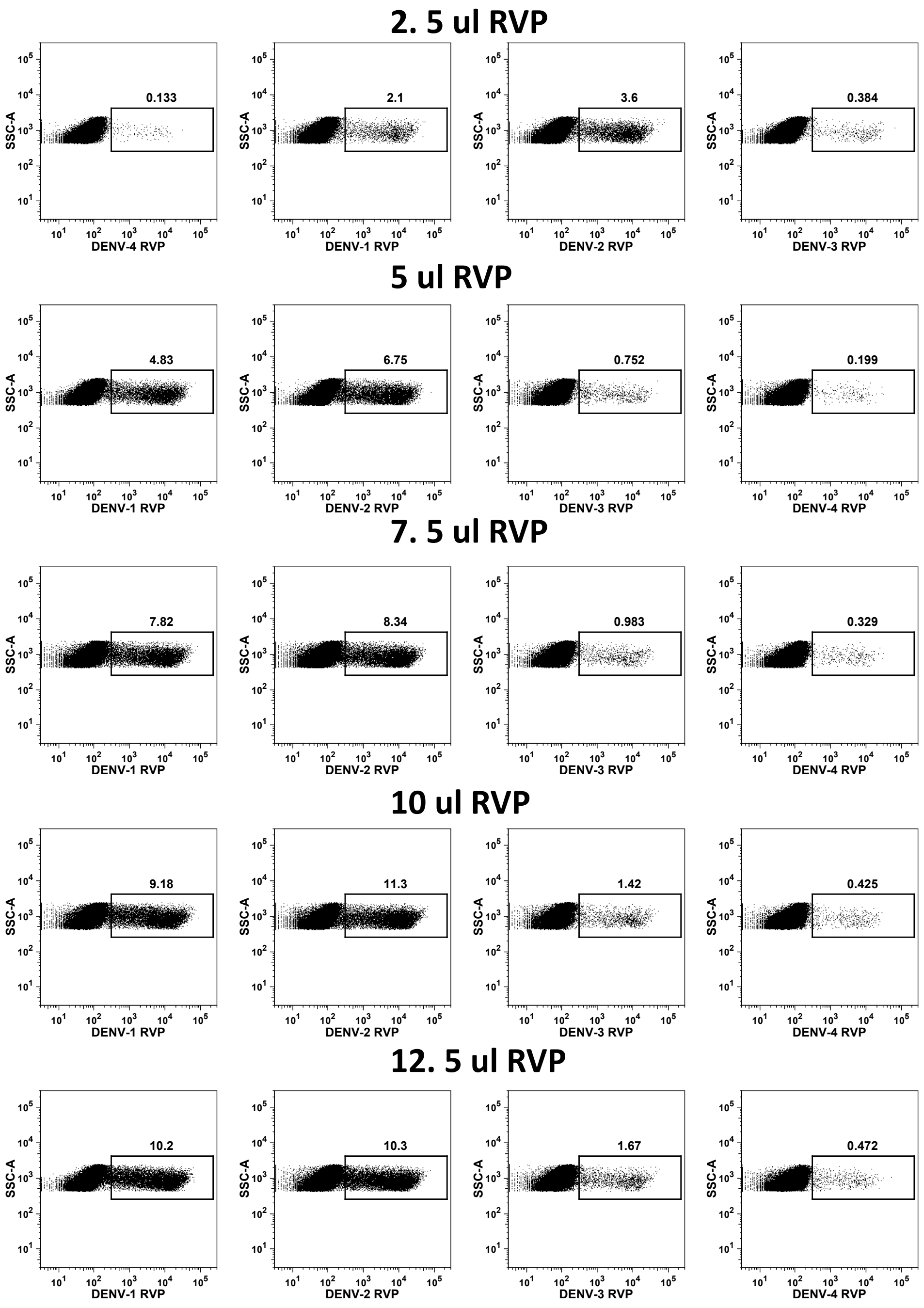
2. 5 ul RVP
0.133
2.1
3.6
0.384
SSC-A
DENV-4 RVP
DENV-1 RVP
DENV-2 RVP
DENV-3 RVP
5 ul RVP
4.83
6.75
0.752
0.199
DENV-1 RVP
DENV-2 RVP
DENV-3 RVP
DENV-4 RVP
7. 5 ul RVP
7.82
8.34
0.983
0.329
DENV-1 RVP
DENV-2 RVP
DENV-3 RVP
DENV-4 RVP
10 ul RVP
9.18
11.3
1.42
0.425
DENV-1 RVP
DENV-2 RVP
DENV-3 RVP
DENV-4 RVP
12. 5 ul RVP
10.2
10.3
1.67
0.472
DENV-1 RVP
DENV-2 RVP
DENV-3 RVP
DENV-4 RVP

Fig. S3

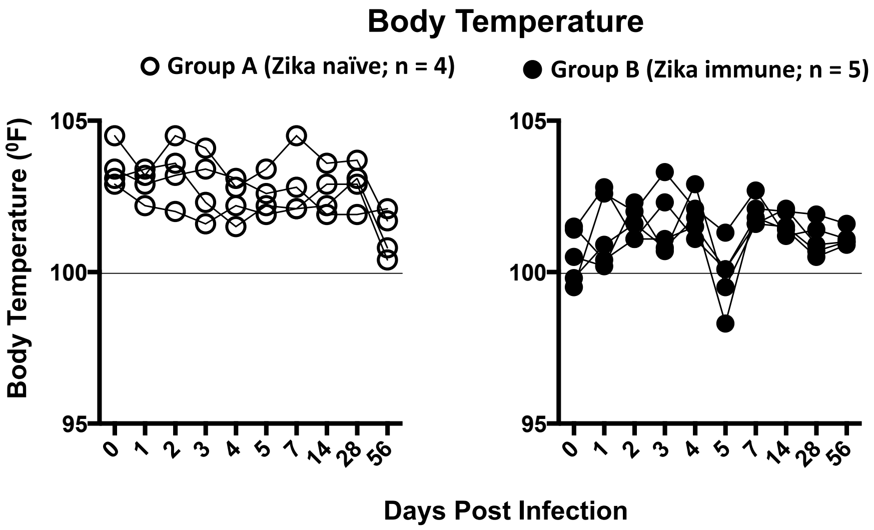

Body Temperature
Group A (Zika naïve; n = 4)
Group B (Zika immune; n = 5)
Body Temperature (⁰F)
105
100
95
0
1
2
3
4
5
7
14
28
56
Days Post Infection

Fig. S4
